# Imported parasitic diseases in mainland China: current status and perspectives for better control and prevention

**DOI:** 10.1186/s40249-018-0454-z

**Published:** 2018-08-03

**Authors:** Lan-Gui Song, Xing-Da Zeng, Yan-Xia Li, Bei-Bei Zhang, Xiao-Ying Wu, Dong-Juan Yuan, Ai He, Zhong-Dao Wu

**Affiliations:** 1 Department of Parasitology, Zhongshan School of Medicine, Sun Yat-sen University, Guangzhou, 510080 Guangdong China; 2 Key Laboratory of Tropical Diseases Control (SYSU), Ministry of Education, Guangzhou, 510080 Guangdong China; 3 Provincial Engineering Technology Research Center for Biological Vector Control, Guangzhou, 510080 Guangdong China; 4 Jiangxi Provincial Testing Center of Medical Apparatus and Instruments, Nanchang, 330029 Jiangxi China; 5 School of Public Health, Fudan University, Shanghai, 200433 China

**Keywords:** Parasitic diseases, Imported disease, China

## Abstract

**Background:**

The high prevalence of parasitic diseases leads to millions of deaths and disabilities each year in developing countries. China has also been greatly affected by parasitic infections, including filariasis, leishmaniasis, malaria, schistosomiasis, and soil-transmitted nematodosis. However, the situation in China improved dramatically after comprehensive parasitic disease control efforts were strengthened, leading to the elimination of filariasis in 2006 and to significant control over other diseases. However, imported parasitic disease cases are inevitable, and such cases have increasingly been reported as a result of enhanced globalization and international or regional cooperation. These imported diseases represent a major obstacle to the elimination of several parasitoses, such as malaria.

**Main text:**

This paper reviews imported cases of parasitic diseases in mainland China, particularly malaria and schistosomiasis, based on data reported separately by the Chinese annual reports and from other published papers. We summarize the new challenges that face parasitic disease control efforts in mainland China and perspectives regarding better control. We argue that both the provision of professional education and updated training for medical care personnel and the management and surveillance of people entering China are essential. We recommend that Chinese migrant workers should be considered a priority group for health education and that public awareness of imported diseases should be emphasized. Furthermore, we underscore the importance of investigating the distribution of introduced/potential vectors, parasite susceptibility, and improvements in diagnostic techniques and drug stocks.

**Conclusions:**

Imported cases have become the main challenge to the elimination of several parasitoses, such as malaria and schistosomiasis, in mainland China. China should act to meet these challenges, which are closely associated with national biological safety.

**Electronic supplementary material:**

The online version of this article (10.1186/s40249-018-0454-z) contains supplementary material, which is available to authorized users.

## Multilingual abstract

Please see Additional file 1 for translations of the abstract into the five official working languages of the United Nations.

## Background

Parasitic infections are considered diseases of poverty and are highly prevalent in developing countries, leading to millions of deaths and disabilities each year [1, 2]. Although humans in China previously suffered significantly from parasitic diseases [2], the rapid economic growth over the past six decades has led to the increased allocation of resources toward the control of parasitic diseases. Consequently, the status of parasitic diseases in China has improved considerably (Table 1) [3–9]. For instance, more than 30 million cases of lymphatic filariasis, a highly disfiguring infectious disease, were reported in 16 provinces/autonomous regions of mainland China during the 1950s. These cases included 5.4 million patients with severe disease who presented with signs such as elephantiasis, chyluria, hydroceles, and vaginal infection. After long-term efforts, China became the first developing country in the world to eliminate this parasitic disease in 2006 [3].

**Table 1:** Previous and current status of the main parasitic diseases in mainland China

Parasitic diseases	Estimated no. of patients		Endemic provinces/autonomous regions		Transmission vector	Transmission route	Elimination program
	Past (million)	Present	Past	Present ^a^			
Filariasis	30 (1950s)	0 (2016)	16	0	Mosquito	Mosquito bites	Done
Leishmaniosis	0.53 (1950s)	400 ± annually ^b^	16	7 ^c^	Sandfly	Sandfly bites	Almost done
Malaria	30 (1960s)	3321 (2016)	24	2 ^d^	*Anopheline* mosquito	Mosquito bites	Ongoing
Schistosomiasis	11.6 (1950s)	54 454 (2016)	12	7 ^e^	*Oncomelania hupensis*	Infested water contact	Starting
Echinococcosis	0.38 (2004)	40 845 (2015)	21	9 ^f^	–	Fecal-oral route	–
Soil-transmitted nematodosis	536 (1990)	3.12% (2013)	31	31 ^g^	–	Fecal-oral route/skin	–

^a^Present endemic province/autonomous regions: local infections

^b^The prevalence of leishmaniosis in mainland China is not available; only annual new cases were found

^c^Seven provinces/autonomous regions: Xinjiang, Gansu, Sichuan, Shanxi, Shaanxi, Neimenggu, Henan

^d^Three provinces/autonomous regions: Yunnan, Tibet

^e^Seven provinces/autonomous regions: Hunan, Jiangxi, Anhui, Hubei, Yunnan, Sichuan, Jiangsu

^f^Nine provinces/autonomous regions: Tibet, Qinghai, Neimenggu, Xinjiang, Shanxi, Gansu, Ningxia, Yunnan, Sichuan

^g^Data for Hong Kong, Macao and Taiwan Province were not available and are not included

Leishmaniasis, or kala-azar disease, has been classified as one of the most neglected tropical diseases by the World Health Organization (WHO) and represents a serious global health burden [4]. Although China reported approximately 0.53 million patients with leishmaniasis in 16 provinces/autonomous regions during the early 1950s, this disease was nearly eliminated in 1958 by the large-scale treatment of infected dogs (reservoir hosts) and the use of antiparasitic drugs [4]. Approximately 400 new cases of this zoonotic parasite occur annually in China [5].

Malaria, which caused 445 000 deaths in 2016, is the most devastating parasitic disease worldwide [10]. More than 24 million people in 24 provinces/autonomous regions of mainland China were estimated to suffer from malaria in the 1970s [11]. Following unrelenting efforts, a Chinese national malaria prevention and control program has progressed to the next stage, the Action Plan of China Malaria Elimination (2010–2020) [12]. Accordingly, malaria elimination has transformed from a concept to a fact, as demonstrated by the cases reported in mainland China during 2016: approximately 99.9% of the cases (3317 of 3321) were imported, whereas only 0.1% of the cases (3 of 3321) involved local infections in border regions such as Yunnan and Tibet [8].

Schistosomiasis is another debilitating parasitic disease with severe symptoms, such as hepatosplenomegaly, liver fibrosis, and upper gastrointestinal bleeding. In the 1950s, schistosomiasis was estimated to affect 11.6 million people in mainland China, and the intermediate snail host, *Oncomelania hupensis*, is found in 14.3 billion square meters across 12 provinces/autonomous regions, leading to an infection risk for more than 100 million people who reside in these areas [7]. However, the number of schistosomiasis cases decreased to 54 454, and the schistosome infection rates of both humans and livestock decreased to less than 1% throughout all 451 endemic counties in 2016 [13]. Accordingly, China is currently working toward the elimination of schistosomiasis and is expected to soon fulfill the goals outlined by the Healthy China 2030 Plan.

Echinococcosis, a natural-focal disease, is costly and complicated to treat because the treatment regimen generally involves extensive surgery followed by prolonged and intensive drug therapy. Estimates suggest that although 380 000 persons acquired this infection in 2004 [14], the number of cases decreased to 40 845 in 2015 following the implementation of the Action Plan for Echinococcosis Control (2010–2015) [9]. Nevertheless, echinococcosis remains challenging in China, and the control program should be continued and expanded.

Soil-transmitted helminthiasis (i.e., infections with roundworms, pinworms, whipworms, and hookworms) is widely distributed throughout China. The prevalence of this condition has been extremely high, with positive rates of up to 53.67% (536 million infections) among individuals tested during the first national survey of human parasitic diseases in China conducted in 1990 [14]. A comprehensive surveillance system and integrated control campaign launched in 2006 have led to a steady and apparent decline in nematodiasis, with average infection rates of 20.88, 18.93, 16.59, 13.30, 11.25, 9.67, 6.90, and 3.12% in 2006, 2007, 2008, 2009, 2010, 2011, 2012, and 2013, respectively [6]. These data demonstrate the remarkable achievements in local parasitic diseases control throughout China and indicate that these efforts toward the prevention and control of parasitic diseases should be continued.

However, new imported cases of parasitic diseases are inevitable and will likely become increasingly common due to enhanced globalization as well as international and regional communication and cooperation. Estimates suggest that as many as 244 million people worldwide were living outside their countries of birth in 2015 [15]. Travel exchanges between different environments play a crucial role in the successful management of parasitic diseases in migrant receiving countries, particularly in cases in which vectors (e.g., snails, mosquitoes) are also present in China and may thus induce local infections.

## Main text

### Review of imported parasitic diseases in mainland China

**Fig. 1:**
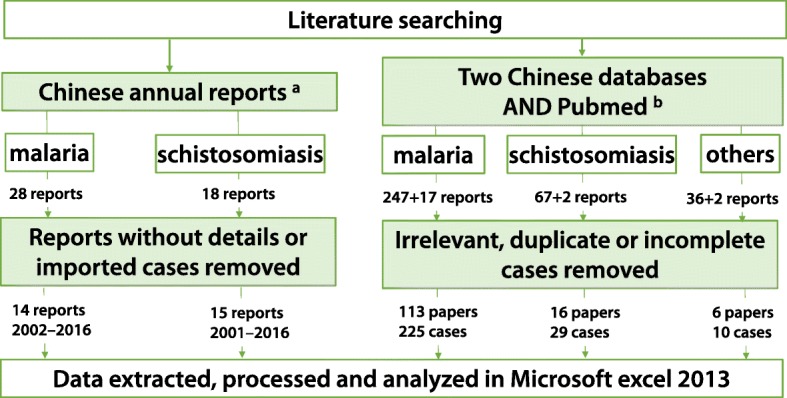
Overall process of reviewing Chinese annual reports and other literature. ^a^ Chinese annual reports: annual *Malaria Situation in the People’s Republic of China* collected for *Chin J Parasitol Parasit Dis* (1988–2016; 2001 was not available) and annual *Endemic status of schistosomiasis in People’s Republic of China* collected from *Chin J Schisto Control* (1999–2016). ^b^ Two (largest) Chinese databases: CNKI and Wanfang; one English database search engine: PubMed

Figure 1 presents the entire literature review process. First, annual reports from the Chinese Center for Disease Control and Prevention (published in *Malaria Situation in the People’s Republic of China* and *Endemic status of schistosomiasis in People’s Republic of China*) were examined. Relevant data (reported province/autonomous regions, source country/province, parasite species) were extracted, processed, and analysed using Excel 2013 (Microsoft Corp., Redmond, WA, USA). Then, a systematic literature search was conducted in CNKI (http://cnki.net/), Wanfang (http://new.wanfangdata.com.cn/) and PubMed (https://www.ncbi.nlm.nih.gov/pubmed) about the imported parasitic diseases in mainland China. The retrieval time: From Jan 1980 to Jan 2018. The keywords “imported and (Malaria or Schistosomiasis or Trypanosomiasis or Loaiasis or Echinococcosis or Soil-transmitted helminthiasis or Filariasis or Leishmaniosis) and China”. All identified papers were screened. Inclusive criteria: (1) Studies that were published from Jan 1980 to Jan 2018; (2) Studies that were published in Chinese or English; (3) Studies that focused on imported parasitic diseases in mainland China. Exclusion criteria: (1) Cases containing overlapping data; (2) Cases without details (age, sex, occupation, travel history, diagnostic process, reported province/autonomous regions, source country/province, parasite species). Relevant data were extracted, processed, and analysed using Excel 2013.

### Current status of imported parasitic diseases

#### Imported malaria

Although malaria was once highly prevalent in 24 provinces/autonomous regions of China, local infections were reported in only Yunnan and Tibet in 2016 [8, 12]. However, except for Tibet, the remaining 30 provinces/autonomous regions in mainland China reported imported cases in the latest Chinese annual report (2016) [8]. The top five provinces/autonomous regions in terms of reported cases imported from abroad (i.e., Chinese residents who acquire an infection abroad and return to China or foreign travellers who acquire an infection in their own country before traveling to China) were Yunnan, Guangxi, Jiangsu, Sichuan, and Henan during 2011 and 2016. The five main source countries of these malarial infections were Myanmar, Ghana, Angola, Equatorial Guinea, and Nigeria during 2011 and 2014 (Table 2) [8, 16–20]. During 2011 and 2016, *Plasmodium falciparum* malaria was most common, causing 61.5% of cases, followed by *P. vivax* malaria (27.8%), *P. ovale* malaria (5.1%) and *P. malariae* malaria (1.4%) (Table 3) [8, 16–20]. One case of *P. knowlesi* malaria was reported in 2014 [19]. Countries that share borders with China, such as Myanmar, continue to suffer intensely from malaria, and this fact presents an obstacle to the elimination of local infections in Chinese border regions [8]. Accordingly, a few cases of domestically mobile (i.e., imported inter- and intra-provincially) infection have been reported annually over the past five years [8, 16–20].

**Table 2:** Five main reported provinces/autonomous regions in mainland China and source countries associated with cases of malaria imported from abroad (Chinese annual report) from 2011 to 2016

Year	No. of reported provinces/autonomous regions	No. of cases from five main reported provinces/autonomous regions					No. of cases from five main source countries				
		YN	GX	JS	SC	HN	Myanmar	Ghana	Angola	Equatorial Guinea	Nigeria
2016	30	410	305	307	327	198	–	–	–	–	–
2015	31	585	236	405	290	184	–	–	–	–	–
2014	30 ^a^	486	184	355	264	216	488	188	278	289	330
2013	30 ^b^	513	1251	341	234	194	608	1345	442	296	217
2012	29 ^c^	679	219	198	148	150	764	241	151	233	197
2011	25	1086	111	357	172	184	1148	88	277	199	243
Total	–	3759	2306	1963	1435	1126	3008	1862	1148	1017	987

YN: Yunan; GX: Guangxi; JS: Jiangsu; SC: Sichuan; HN: Henan

Mainland China has 31 provinces (Hong Kong, Macao, and Taiwan Province not included)

^a^Except for Neimenggu; ^b^ except for Tibet; ^c^ except for Tibet and Qinghai

**Table 3:** Numbers of cases (Chinese annual report) of each type of malaria imported from abroad in mainland China from 2011 to 2015

Diagnosis year	No. of cases	*P. falciparum* malaria		*P. vivax* malaria		*P. ovale* malaria		*P. malariae* malaria		Mixed infection		Unidentified type		*P. knowlesi* malaria	
		No.	%	No.	%	No.	%	No.	%	No.	%	No.	%	No.	%
2016	3317	2158	65.1	709	21.4	311	9.4	64	1.9	61	1.8	14	0.4	0	0.0
2015	3248	1991	61.3	851	26.2	272	8.4	70	2.2	47	1.5	17	0.5	0	0.0
2014	3021	1876	62.1	798	26.4	231	7.7	52	1.7	44	1.5	19	0.6	1	0.0
2013	4042	2899	71.7	859	21.2	133	3.3	51	1.3	65	1.6	35	0.9	0	0.0
2012	2474	1403	56.7	901	36.4	28	1.1	28	1.1	39	1.6	75	3.0	0	0.0
2011	2974	1414	47.6	1183	39.8	0	0.0	0	0.0	99	3.3	278	9.4	0	0.0
Total	19 076	11 741	61.5	5301	27.8	975	5.1	265	1.4	355	1.9	438	2.3	1	0.0

The literature reports of imported malaria cases are not fully consistent with those reported by the Chinese annual report [21–111]. For instance, Yunnan Province reported most of the imported cases in the past consecutive three annual reports (2014–2016), but Hubei Province reported most of the imported cases in the other literature reports from 1986 to 2017, and the proportion of *P. falciparum* malaria was higher in literature reports (75%) than in the Chinese annual report (65% in 2016). Both the literature and Chinese annual reports described similar source countries, and both ranked Myanmar first. Among the 113 imported malaria cases from literature from 1986 to 2017, 108 (95.6%) were identified in men; this was likely because 73 of the cases (64.6%) involved migrant workers from Africa or Southeast Asia from 2000 to 2017. Twelve deaths (10.6%) were reported and attributed to misdiagnosis or delayed treatment from 1989 to 2013. Only a few domestically mobile cases were reported, and all occurred at least ten years ago [112–124].

#### Imported schistosomiasis

Of the five main types of schistosomiasis, only schistosomiasis japonica is prevalent in China [7]. Transmission of this disease has already ceased in six (Sichuan, Zhejiang, Shanghai, Fujian, Guangdong, and Guangxi) of the original 12 endemic provinces/autonomous regions, but cases continue to occur in Yunnan, Jiangsu, Hubei, Anhui, Hunan, and Jiangxi [7, 125]. Therefore, schistosomiasis japonica remains domestically mobile in China. A summary of imported cases of schistosomiasis (Chinese annual reports) in mainland China from 2001 to 2016 is provided in Additional file 2 [13, 126–140]. Only four cases of schistosomiasis mansoni imported from abroad were reported in Beijing (2008, 2010) and Zhejiang (2013, 2015), and two cases of schistosomiasis haematobia were reported in Zhejiang in 2016. Overall, 123 chronic and 65 acute imported cases of schistosomiasis japonica were reported during 2011 and 2016 and were mainly distributed in Zhejiang and Shanghai.

The relevant literature from 1999 to 2016 is displayed in Table 4. Nine schistosomiasis cases imported from abroad have been reported, of which eight involved Chinese migrant workers returning from Africa (Angola, Nigeria and Tanzania) and the ninth involved a businessman returning from Ghana [141–148]. Twenty domestically mobile cases were reported in mainland China [149–155], fifteen of which involved transmission from non-endemic or transmission-disrupted areas to endemic areas wherein patients were infected upon returning to their hometowns. The remaining five patients were migrant workers from endemic areas in Zhejiang (a transmission-disrupted province).

**Table 4:** Domestically mobile cases of schistosomiasis and those imported from abroad in mainland China from 1999 to 2016 (literature-based)

Diagnosis year	Reported province	Sex	Age	Source country/province	Activity/occupation	Diseases ^c^	Ref
2016	Fujian	1 M	30	Angola	Migrant worker ^a^	Sh	141
2015	Shandong	1 M	38	Angola	Migrant worker ^a^	Sh	142
2015	Zhejiang	1 M	52	Nigeria	Migrant worker ^a^	Sh	143
2015	Zhejiang	1 M	53	Nigeria	Migrant worker ^a^	Sh	144
2013	Zhejiang	1 M	46	Nigeria	Migrant worker ^a^	Sm	145
2012	Shanxi	1 M	36	Angola	Migrant worker ^a^	Sh	146
2012	Fujian	1 M	25	Ghana	businessman	Sh	147
2011	Henan	1 M	40	Angola	Migrant worker ^a^	Sh	148
2011	Henan	1 M	33	Tanzania	Migrant worker ^a^	Sh	148
2009	Jiangsu	1 M 1F	9	Jiangxi	Student	Sj	149
2005	Guizhou	8 M	30–54	Hubei/Hunan	Catch birds	Sj	150
2005	Zhejiang	1 M	18	Anhui	Migrant worker ^b^	Sj	151
2004	Zhejiang	1 M	49	Hubei	Migrant worker ^b^	Sj	151
2004	Zhejiang	1F	41	Sichuan	Migrant worker ^b^	Sj	152
2003	Zhejiang	1 M	36	Anhui	Migrant worker ^b^	Sj	153
2003	Zhejiang	1 M	24	Hubei	Migrant worker ^b^	Sj	153
2000	Guangdong	1 M	19	Hunan	Shipbuilder	Sj	154
1999	Zhejiang	1 M	39	Anhui	Fish farming	Sj	155

*M* Male, *F* Female

^a^Migrant worker to Africa; ^b^Migrant worker to another province/autonomous regions in mainland China

^c^Sh: Schistosomiasis haematobia; Sm: Schistosomiasis mansoni; Sj: Schistosomiasis japonica

#### Other imported parasitic diseases

A literature-based list of imported parasitic diseases other than malaria and schistosomiasis in mainland China is presented in Table 5 [156–161].

**Table 5:** Abroad-imported cases of other parasitic diseases in mainland China (literature-based)

Diagnosis year	Reported province/autonomous regions	Sex	Age	Source country	Activity/occupation	Diseases	Ref
2017	Fujian	F	41	Kenya/Tanzania	Travel	Trypanosomiasis	156
2015	Beijing	M	49	Congo	Migrant worker	Loaiasis	157
2015	Beijing	M	28	Nigeria	Cartwright	Loaiasis	158
2015	Beijing	M	49	Congo	Migrant worker	Loaiasis	158
2014	Jiangsu	M	45	Gabon	Migrant worker	Trypanosomiasis	159
2013	Beijing	M	33	Gabon/Cameroun	Migrant worker	Loaiasis	158
2012	Beijing	M	25	Gabon	Engineer	Loaiasis	158
2012	Beijing	M	23	Cameroun	Translator	Loaiasis	158
2008	Zhejiang	M	45	Gabon	Farming	Loaiasis	160
2008	Zhejiang	M	45	Gabon	Migrant worker	Loaiasis	161

*M* Male, *F* Female

### Perspectives for better control and prevention

Estimates suggest that as many as 244 million people worldwide are living outside their country of birth [15], and travel between different environments plays a crucial role in the management of parasitic diseases in countries that receive migrants. In other words, imported parasitic diseases are unavoidable. The China Africa Project, which aimed to assist with infrastructure construction, has led to a dramatic increase in the number of migrant workers who have moved to Africa since the 1970s [7]. In addition, increasing numbers of Chinese companies, particularly the communications industries, have established overseas subsidiaries over the past decade to capitalize on the African market. Current estimates indicate that more than 1 million Chinese live in Africa [7]. These individuals lack knowledge about self-protection and are at risk of acquiring schistosomiasis while swimming in unclean water, developing malaria after failing to protect against mosquito bites, or contracting other parasitic infections by eating infected food. This situation is exacerbated by a failure to report their travel history to a physician. Because many physicians in China are somewhat unfamiliar with parasitology, this omission presents an obstacle to a correct diagnosis [162]. The Belt and Road Initiative, proposed in 2013 by Chinese president Xi Jinping, aimed to build a trade and infrastructure network that would connect Asia with Europe and Africa along the land-based Silk Road Economic Belt and the ocean-based Maritime Silk Road. More than 100 countries and international organizations are or will be involved in this program, which will affect nearly two-thirds of the global population and approximately a quarter of all goods and services needed for global functioning [163]. Undoubtedly, however, this program will present new challenges to Chinese parasitic disease control programs. The detection of imported cases and the provision of prompt treatment will be essential to reducing the risk of an outbreak of parasitic disease, particularly of nonindigenous parasites, as the establishment of a new parasite may lead to catastrophic consequences. Therefore, universal participation in the campaign against imported parasitic diseases in mainland China is required and includes the transmission of information regarding health care organizations (clinics and hospitals), disease control and prevention centers (CDC), governmental public health departments, entry and exit administrations, labour service companies, transnational corporations, and tourist administrations.

#### Strengthening the essentiality of professional education and updated training for medical care personnel

The parasitic disease control program in China has made impressive progress, and its status is particularly important, particularly with regard to the rapidly developing economy in mainland China. This program is especially essential to highly developed areas such as Guangdong, Shanghai, and Beijing, where fewer resources are available to communicate knowledge about parasitic diseases [162]. In the absence of sufficient financial resources, general practitioners and specialists might no longer be equipped with sufficient parasitological knowledge. Accordingly, primary health care providers might misdiagnose many cases of malaria as a cold or flu, leading to delayed treatment [26, 45, 64, 70, 79, 81]. As a result, some patients might not receive an accurate diagnosis even after referral to a specialist [57, 63, 68, 71], and the resulting treatment delay may be lethal for patients infected with *P. falciparum*.

Furthermore, technicians may not be able to differentiate *Plasmodium* species in blood smears [26, 34, 42, 94, 105], especially in small hospitals without DNA detection-related equipment, and patients with *P. falciparum* co-infection have died after receiving a diagnosis of the less-serious *P. vivax* malaria [20, 105]. A recent report describes the case of a 36-year-old male migrant worker who was misdiagnosed with a ureteral calculus, invasive urothelial carcinoma, and eosinophilic cystitis at several hospitals after returning from Angola. The patient underwent several treatments, including transurethral bladder tumor resection, before finally receiving a definite diagnosis of schistosomiasis haematobia after a pathology examination revealed eggs in the bladder tissue [146]. The eventual diagnosis occurred one year after his first visit to the clinic, and his subsequent experience had both physical and mental effects. Similarly, more than two months were required to receive a final diagnosis in the first case of human African trypanosomiasis in China. During this delay, the trypanosome parasites were able to cross the blood-brain barrier and enter the spinal fluid to infect the central nervous system, leading to irreversible damage [159]. Although imported cases are difficult to diagnose, much can be done to avoid future tragedies. Among medical care personnel, the awareness of parasitic diseases and parasitological knowledge must be improved. Professional parasitological education should be strengthened, rather than neglected, in medical schools. Furthermore, regular training courses are needed to ensure that relevant medical personnel receive updated knowledge and improve their expertise.

#### Putting Chinese migrant workers as a priority group for health education and public awareness of imported diseases

As mentioned above, the number of Chinese migrant workers is expected to increase rapidly to meet the goals of large Chinese governmental programs such as “Aid to Africa” and the Belt and Road Initiative. Most migrant workers will participate in outdoor work projects, including the construction of water reservoirs, electric power stations, and roads and in mining and oil exploitation. These workers tend to have a lower socioeconomic status and education level and are thus less likely to possess adequate health knowledge. Accordingly, they are vulnerable to environmental risk factors such as contact with unsafe water and insect bites and are therefore very susceptible to parasitic infection. Therefore, labour service companies must conduct health education activities, distribute medicine, and provide necessary personal protective equipment to the migrant workers before they leave China. Governmental health departments should also provide guidance and supervision during this process. In practice, however, these procedures fall short of the targets. Some returnees understand that they might have developed malaria if they have a fever, and this knowledge can help the physician make a timely diagnosis [81]. More commonly, however, the returnee does not know what type of disease he or she might have acquired, and the connection between the disease and health risk behaviour remains hidden. Furthermore, some returnees do not report their travel history to the physician, which contributes significantly to misdiagnosis [75, 101]. Accordingly, more resources should be allocated to the health education of prospective migrant workers. For example, CDCs could assign professionals to deliver informative lectures to those workers. Other people, such as businessmen, students, technicians, and travelers, are also susceptible to parasitic infection. Therefore, information departments and tourist administrations should also raise awareness of the potential dangers by distributing brochures, displaying posters, and playing promotional videos.

#### Intensifying the management and surveillance of people entering China (international and domestic passengers)

Imported parasitic diseases are an inevitable and increasingly common consequence of globalization, as well as international and regional communications and cooperation. Issues related to the management and surveillance of people entering China are broad, high-level, and cross-cutting. Travel between different environments will inevitably result in the importation of parasitic disease agents and the introduction of new parasites to China. In the absence of a sound management and surveillance system, such travel might lead to the formation of an endemic focus, especially if the parasite vector (snail, mosquito) is present in China.

The number of domestic travellers continues to grow steadily, as demonstrated by the increasing number of Chinese travellers to other countries (101, 117, and 122 million person-times in 2014, 2015, and 2016, respectively) [164–166]. The number of international visitors (and compatriots from Hong Kong, Macao, and Taiwan Province) has also increased (128, 134, and 138 million persons in 2014, 2015, and 2016, respectively) [164–166]. While traveling, domestic passengers might acquire certain infections because of a lack of knowledge about transmission and a lack of specific antibodies. These factors are especially important for travelers who visit Africa and Southeast Asia and for international visitors from endemic areas who may have already developed or will develop the disease. Thus, entry inspection plays a vital role in the management of such potential infection sources. The CDC staff should monitor the health service usage and health outcomes of suspected or confirmed cases of a certain parasitic diseases. For example, the condition of a 43-year-old migrant worker with falciparum malaria worsened after a failure to comply with medical instructions provided by the CDC staff [44]. The development of management and surveillance guidelines, particularly for non-endemic parasitic diseases, is increasing in importance.

#### Increasing investigation of the distribution of introduced/potential vectors and susceptibility to parasites

The Chinese population is increasingly at risk of contracting parasitic diseases that are not indigenous to China as a result of the return of infected travelers or the arrival of infected foreigners [167, 168]. The risk of transmission of such diseases increases when the parasite vectors are present in or have been introduced to China. The natural *S. mansoni* vector, *Biomphalaria straminea*, was found in Hong Kong in 1974. In 2013, its distribution was found to have expanded to nearby cities such as Shenzhen, Dongguan, and Huizhou, Guangdong, and it might since have spread via waterways in Guangdong [7]. Because the Guangdong region is characterized by many rivers and frequent international communications, the parasite’s life cycle may be established by the introduction of patient fecal matter into water containing *Biomphalaria* snails. This would be a matter of great public concern. For other nonindigenous parasitic diseases such as Chagas disease (*Trypanosoma cruzi*), the vector (triatomine insects) was found to be distributed throughout China [169], although the susceptibility of the vector to *T. cruzi* remains unclear. Therefore, it is important to understand both the distribution of the vectors and their susceptibilities to parasites. The CDC staff and scientific researchers should collaborate to conduct relevant field investigations and laboratory research.

#### Improving diagnostic techniques and drug stocks

 Many parasitic diseases are clinically curable if treatment is initiated at an early stage. Therefore, universal access to rapid diagnosis and care is essential. However, the diagnosis of an imported parasitic disease is complex and requires specialized staff, because many diagnostic techniques are far from satisfactory. For example, the current rapid diagnostic tests for malaria are somewhat unreliable, and repeated false-negative cases [46, 61, 86] have led to multiple missed diagnoses at ports of entry and community health care centres. Although the microscopic examination of a peripheral blood smear is currently the gold standard for malaria diagnosis, this technique is limited by a high false-negative rate [41, 84], especially in cases with low degrees of parasitemia. Furthermore, it is difficult to differentiate among *Plasmodium* species, and coinfections are often missed [27]. Although DNA detection techniques have been developed to overcome these limitations [21], these methods are much more expensive, and each small clinic cannot realistically be supplied with an expensive PCR amplifier and skilled technicians. Accordingly, early, rapid, economically feasible, and accurate diagnostic methods are in desperate demand. Foundations should support studies in this field and encourage researchers to conduct related studies. In addition, because the rapid diagnosis of an imported parasitic disease is meaningless without prompt treatment, access to therapeutic interventions for rare parasitic diseases is also a significant factor. The CDC should establish a secure drug supply system to guarantee that patients with rare infections can obtain timely therapeutic interventions.

## Conclusions

In the context of globalization, which is underscored by large Chinese governmental programs such as “Aid to Africa” and the “Belt and Road Initiative”, the Chinese parasitic disease control program will face new challenges. For example, imported cases of disease represent a major obstacle to the elimination of several parasitoses such as malaria and schistosomiasis, and some nonindigenous pathogens (and their vectors) have emerged in mainland China. Therefore, China should act to meet these challenges, which are closely associated with national biological safety.

## Additional files

Additional file 1: Multilingual abstracts in the five official working languages of the United Nations. (PDF 349 kb)

Additional file 2: Chinese annual report-based details of imported cases of schistosomiasis in mainland China from 2001 to 2016. (PDF 99 kb)

## Abbreviations

CDC: Centers for Disease Control and Prevention
