# Supplementary material for: Imported parasitic diseases in mainland China: current status and perspectives for better control and prevention
Source: Infect Dis Poverty. 2018 Aug 3;7:78. doi: 10.1186/s40249-018-0454-z (PMC6091017; doi:10.1186/s40249-018-0454-z)
Supplement: Supplementary file 1 — Multilingual abstracts in the five official working languages of the United Nations. (PDF 349 kb) [file 40249_2018_454_MOESM1_ESM.pdf]

Translation of the abstract into the five official working languages of the United Nations

الأمراض الطفيلية الوافدة في برالصين الرئيسي: الوضع الحالي ووجهات النظرلمكافحة ووقاية أفضل

لان غوي سونغ ، شينغ دا تشن ، يان شيا لي ، بيي تشانغ ، شياو يينغ وو ، دونغ جوان يوان ، اي هي وتشونغ داو وو.

الملخص
الخلفية: في البلدان النامية ، يؤدي الانتشار المرتفع للأمراض الطفيلية إلى ملايين الوفيات والإعاقات كل عام. لقد تأثرت الصين وبشدة بالعدوى الطفيلية ، بما في ذلك داء الفيلاريات وداء الليشمانيات , والملاريا , والبلهارسيا والديدان الخيطية المنقولة بالتربة. ومع ذلك ، تحسن الوضع في الصين بشكل كبير بعد تعزيز جهود مكافحة الأمراض الطفيلية الشاملة ، مما أدى إلى القضاء على داء الفيلاريات في عام 2006 وإلى السيطرة الكبيرة على أمراض أخرى. ومع ذلك ، فإن حالات الأمراض الطفيلية الوافدة أمر لا مفر منه ، وقد تم الإبلاغ عن مثل هذه الحالات بصورة متزايدة نتيجة لتعزيز العولمة والتعاون الدولي أو الإقليمي. تمثل هذه الأمراض الوافدة عقبة رئيسية أمام القضاء على العديد من حالات الطفيليات ، مثل الملاريا.
الجزء الأساسي: تستعرض هذه المقالة الحالات الوافدة للأمراض الطفيلية في برالصين الرئيسي ، ولا سيما الملاريا والبلهارسيا ، استناداً إلى البيانات الواردة بشكل منفصل عن التقارير السنوية الصينية ومن الصحف الأخرى المنشورة. نقوم بتلخيص التحديات الجديدة التي تواجه جهود مكافحة الأمراض الطفيلية في بر الصين الرئيسي ووجهات النظر فيما يتعلق بسيطرةٍ أفضل. ونحن نحاول التأكيد على أن توفير التعليم المهني والتدريب المحدث لموظفي الرعاية الطبية وإدارة ومراقبة الأشخاص الذين يدخلون الصين أمران أساسيان. وعلاوة على ذلك ، نوصي بأن يتم اعتبار العمال المهاجرين الصينيين مجموعة ذات أولوية للتثقيف الصحي والتأكيد على الوعي العام بالأمراض الوافدة. وعلاوة على ذلك ، نؤكد على أهمية التحقق من انتشار النواقل الدخيلة / المحتملة ، والقابلية الطفيلية ، والتحسينات في تقنيات التشخيص ومخزونات العقاقير.
الاستنتاجات : أصبحت الحالات الوافدة هي التحدي الرئيسي أمام القضاء على العديد من حالات الطفيليات ، مثل الملاريا والبلهارسيا ، في برالصين الرئيسي. يجب أن تعمل الصين على مواجهة هذه التحديات ، التي ترتبط ارتباطًا وثيقًا بالسلامة البيولوجية الوطنية.

Translated from English version into Arabic by Rand Gharaibeh and Free bird, through

TRANSLATORS
WITHOUT BORDERS

中国大陆输入性寄生虫病的现状与防控策略

宋兰桂，曾幸达，李雁霞，张蓓蓓，吴晓瑛，元冬娟，何 蔼，吴忠道

摘要

引言：在发展中国家，寄生虫病的患病率很高，每年导致数百万人残疾与死亡。中国也曾饱受寄生虫感染的折磨，包括丝虫病、利什曼病、疟疾、血吸虫病和土源性的线虫病。然而，随着综合性寄生虫病防治工作的深入推进，中国的情况大幅改善，比如 2006 年消除了丝虫病，并对其他寄生虫病进行了有效的控制。但是，随着全球化和国际或区域合作的不断加强，输入性寄生虫病是无法避免的，相关的病例报道也越来越多。这些输入性疾病将是消除其他寄生虫病的主要障碍，比如疟疾。

主体：本文根据中国年度报告和其他相关的出版论文回顾了中国大陆输入性寄生虫病例的情况，特别是疟疾和血吸虫病。在寄生虫病防治工作方面，我们总结了中国大陆面临的新挑战以及相应的防控策略。我们认为，提供专业的教育和定期培训给医护人员以及对加强对进

入中国人员的管理与监测是至关重要的。同时，我们建议中国外派务工人员应该被视为健康教育的重点群体，以及应加强公众对输入性疾病的认识。此外，我们强调对入侵/潜在媒介的分布调查与寄生虫易感性研究以及诊断技术与药物库存改进的重要性。
结论：输入性病例已成为中国大陆消除几种寄生虫病（如疟疾和血吸虫病）的主要挑战。中国应采取行动应对这些与国家生物安全密切相关的挑战。

Translated from English version into Chinese by Lan-Gui Song

Parasitoses importées en Chine continentale : état des lieux et perspectives d'amélioration de la lutte et de la prévention

Lan-Gui Song, Xing-Da Zeng, Yan-Xia Li, Bei-Bei Zhang, Xiao-Ying Wu, Dong-Juan Yuan, Ai He et Zhong-Dao Wu

Résumé

Contexte : Dans les pays en voie de développement, la forte prévalence des parasitoses provoque chaque année des millions de morts et d'infirmités. La Chine a été, elle aussi, fortement affectée par de nombreuses maladies parasitaires : filariose, leishmaniose, paludisme, schistosomiase et géohelminthiases. Sa situation s'est cependant améliorée de façon spectaculaire après la montée en puissance des efforts de lutte contre les parasitoses, qui a permis d'éliminer la filariose en 2006 et d'endiguer significativement les autres maladies parasitaires. Malgré cela, les cas de parasitoses importées sont inévitables et, à cause de la mondialisation accrue et de la coopération internationale ou régionale, ils deviennent de plus en plus fréquents. Ces maladies importées représentent un obstacle de taille à l'élimination de plusieurs maladies parasitaires telles que le paludisme.
Discussion : Cet article passe en revue les cas de maladies parasitaires importées en Chine continentale, notamment de paludisme et de schistosomiase, à partir des données fournies par les rapports annuels chinois et d'autres articles publiés. Nous y résumons les nouveaux obstacles aux efforts de lutte contre les parasitoses en Chine continentale et les perspectives d'amélioration. Nous soutenons que la formation professionnelle, la mise à jour des connaissances des professionnels de la santé ainsi que l'encadrement et la surveillance des personnes entrant en Chine sont d'une importance cruciale à cet égard. Nous suggérons en outre que les travailleurs migrants chinois reçoivent en priorité une éducation sanitaire et que le public soit encore davantage sensibilisé aux maladies importées. Par ailleurs, nous soulignons l'importance d'enquêter sur la propagation de vecteurs introduits ou potentiels, sur la susceptibilité aux infections parasitaires et sur l'amélioration des techniques de diagnostic et des stocks de médicaments.
Conclusions : Les cas importés sont devenus le principal obstacle à l'élimination de plusieurs parasitoses telles que le paludisme et la schistosomiase en Chine continentale. La Chine doit agir afin de surmonter ces obstacles, étroitement liés à sa sécurité biologique nationale.

Translated from English version into French by William Squire and Suzanne Assenat, through

Завезённые паразитарные болезни в континентальном Китае: текущее положение и перспективы по улучшению контроля и профилактики

Лань-Гуй Сун, Син-Да Цзэн, Янь-Ся Ли, Бэй-Бэй Чжан, Сяо-Ин У, Дун-Цзюань Юань, Ай Хэ и Чжун-Дао У

Аннотация

Справочная информация

Высокая заболеваемость паразитарными болезнями в развивающихся странах ежегодно приводит к миллионам смертей и инвалидностей. Китай также существенно пострадал от паразитарных инфекций, в том числе от филяриатоза, лейшманиоза, малярии, шистосомоза, а также передаваемых через почву нематодоз. Однако в результате укрепления усилий по всеобъемлющему контролю за паразитарными болезнями ситуация в Китае значительно улучшилась, что привело к искоренению филяриатоза в 2006 году и к существенному контролю за другими заболеваниями. Тем не менее, случаи завезённых паразитарных болезней являются неизбежными, а в результате растущей глобализации и регионального сотрудничества всё чаще поступает информация о таких случаях. Данные завезённые болезни представляют собой основное препятствие на пути устранения нескольких паразитарных заболеваний, таких как малярия.

Основная часть

В настоящей работе рассматриваются завезённые случаи паразитарных болезней в континентальном Китае, в особенности малярии и шистосомоза, на основании данных, отдельно представленных в ежегодных обзорах по Китаю, а также в других научных публикациях. Мы подвели итоги по новым сложностям, с которыми сталкиваются усилия по контролю за паразитарными болезнями в континентальном Китае, а также по перспективам относительно улучшения контроля. Мы считаем, что необходимы как обеспечение профессионального обучения, так и обновлённая подготовка медицинского персонала, а также управление и надзор за лицами, въезжающими в Китай. Более того, мы предлагаем считать китайских трудовых мигрантов приоритетной группой для целей медико-санитарного просвещения, а также подчёркивать необходимость информирования общественности относительно завезённых болезней. Кроме того, мы особо отмечаем важность изучения распространения представленных/потенциальных переносчиков заболеваний, восприимчивости к паразитам, а также улучшения методов диагностики и запасов лекарственных препаратов.

Выводы

Завезённые случаи представляют собой основную сложность на пути к искоренению нескольких паразитарных заболеваний, таких как малярия и шистосомоз, в континентальном

Китае. Китай должен направить свои действия на устранение указанных сложностей, которые тесно связаны с общенациональной биологической безопасностью.

Translated from English version into Russian by Liudmila Tomanek and Natalia Potashnik, through

TRANSLATORS
WITHOUT BORDERS

Enfermedades parasitarias importadas en China continental: Estado actual y perspectivas de mejora en control y prevención.

Lan-Gui Song, Xing-Da Zeng, Yan-Xia Li, Bei-Bei Zhang, Xiao-Ying Wu, Dong-Juan Yuan, Ai He and Zhong-Dao Wu

Resumen

Antecedentes: En países en vía de desarrollo, la alta prevalencia de enfermedades parasitarias provoca millones de muertes y discapacidades cada año. China se ha visto muy afectada por las infecciones parasitarias, incluyendo filariasis, leishmaniasis, malaria, esquistosiomasis y nematodosis transmitida por contacto con el suelo. No obstante, la situación en China ha mejorado drásticamente después de haber reforzado las amplias medidas de control de enfermedades parasitarias, que llevó a eliminar la filariasisen 2006 y a un control significativo de otras enfermedades. Sin embargo, no se pueden evitar los casos de enfermedad parasitaria importada y se han declarado estos casos como resultado de un aumento de la globalización y la cooperación internacional o regional. Estas enfermedades importadas representan un obstáculo importante para la eliminación de diversas parasitoris, como la malaria.

Cuerpo principal: Este artículo revisa casos importados de enfermedades parasitarias en China continental, en particular la malaria y la esquistosiomasis, basado en los datos declarados separadamente por los informe anuales chinos y otros documentos publicados. Resumimos los nuevos retos a los que se enfrentan los controles de enfermedades parasitarias en China continental y las perspectivas en lo que respecta a mejorar el control. Argumentamos que son esenciales tanto la provisión de educación profesional y actualización de la formación de personal sanitario y la gestión y vigilancia de las personas que entren en China. Asimismo, recomendamos que los trabajadores migrantes chinos deberían considerase un grupo prioritario para la educación sanitaria y que el conocimiento público de las enfermedades importadas debería enfatizarse. Además, subrayamos la importancia de la investigación de la distribución de portadores introducidos/potenciales, la susceptibilidad parasitaria y la mejora en técnicas de diagnóstico y el almacenamiento de medicamentos.

Conclusionnes: Los casos importados se han convertido en el desafío más importante para la eliminación de diversas parasitosis, como la malaria y la esquistosiomasis, en China continental. China debe actuar para afrontar estos retos, que se han asociado estrechamente a la seguridad biológica nacional.

Translated from English version into Spanish by María Galonce Terán and Natalia Victoria Gómez, through

TRANSLATORS
WITHOUT BORDERS
