# Supplementary material for: Imported parasitic diseases in mainland China: current status and perspectives for better control and prevention
Source: Infect Dis Poverty. 2018 Aug 3;7:78. doi: 10.1186/s40249-018-0454-z (PMC6091017; doi:10.1186/s40249-018-0454-z)
Supplement: Supplementary file 2 — Chinese annual report-based details of imported cases of schistosomiasis in mainland China from 2001 to 2016. (PDF 99 kb) [file 40249_2018_454_MOESM2_ESM.pdf]

Additional file 2 Summary of imported schistosomiasis (Chinese government report-based) in mainland China during 2001-2016

Diagnosis year	Abroad-imported cases			Domestically-mobile cases (Sj) [c]																
	No. chronic cases	No. acute cases	Reported province [c]	No. chronic cases	Reported provinces			No. acute cases	Reported provinces											
					ZJ	SH	FJ		ZJ	SH	JS	AH	GZ	FJ	SC	GD	BJ	CQ	GX	HN
2016	2	0	ZJ [a]	9	7	2	0	0	0	0	0	0	0	0	0	0	0	0	0	0
2015	1	0	ZJ [b]	7	7	0	0	0	0	0	0	0	0	0	0	0	0	0	0	0
2014	0	0	/	10	9	1	0	0	0	0	0	0	0	0	0	0	0	0	0	0
2013	0	1	ZJ [b]	14	13	1	0	0	0	0	0	0	0	0	0	0	0	0	0	0
2012	0	0	/	8	6	2	0	10	1	0	3	5	0	0	0	0	0	1	0	0
2011	0	0	/	9	7	2	0	3	2	1	0	0	0	0	0	0	0	0	0	0
2010	0	1	BJ [b]	12	9	3	0	3	0	0	2	0	0	0	0	0	1	0	0	0
2009	0	0	/	20	19	1	0	5	1	0	2	0	0	0	0	0	1	0	1	0
2008	1	0	BJ [b]	15	11	2	2	4	2	0	1	0	0	0	1	0	0	0	0	0
2007	0	0	/	25	23	2	0	8	1	1	1	0	0	3	1	1	0	0	0	0
2006	0	0	/	33	31	2	0	3	1	1	0	0	0	1	0	0	0	0	0	0
2005	0	0	/	41	37	4	0	11	2	0	0	0	5	0	1	2	0	0	0	1
2004	0	0	/	14	8	6	0	11	8	3	0	0	0	0	0	0	0	0	0	0
2003	0	0	/	1	0	1	0	4	2	2	0	0	0	0	0	0	0	0	0	0
2002	0	0	/	0	0	0	0	0	0	0	0	0	0	0	0	0	0	0	0	0
2001	0	0	/	0	0	0	0	3	0	3	0	0	0	0	0	0	0	0	0	0
**Total**	**4**	**2**	**/**	**123**	**94**	**27**	**2**	**65**	**20**	**11**	**9**	**5**	**5**	**4**	**3**	**3**	**2**	**1**	**1**	**1**

[a] Sh: schistosomiasis haematobia; [b] Sm: schistosomiasis mansoni; [c] Sj: schistosomiasis japonica

[C] Transmission-disrupted provinces: Zhejiang (ZJ), Shanghai (SH), Fujian (FJ), Guangdong (GD), Guangxi (GX) and Sichuan (SC); Transmission-controlled provinces: Jiangsu (JS) and Anhui (AH); Non-endemic provinces: Guizhou (GZ), Beijing (BJ), Chongqing (CQ) and Henan (HN)
